# Prevalence and correlates of alcohol and other substance use disorders in young adulthood: A population-based study

**DOI:** 10.1186/1471-244X-9-73

**Published:** 2009-11-19

**Authors:** Antti Latvala, Annamari Tuulio-Henriksson, Jonna Perälä, Samuli I Saarni, Terhi Aalto-Setälä, Hillevi Aro, Tellervo Korhonen, Seppo Koskinen, Jouko Lönnqvist, Jaakko Kaprio, Jaana Suvisaari

**Affiliations:** 1 Department of Mental Health and Substance Abuse Services, National Institute for Health and Welfare, Mannerheimintie 166, FIN-00300, Helsinki, Finland; 2 Department of Psychology, University of Helsinki, Finland; 3 Department of Child Psychiatry, Hospital for Children and Adolescents, Helsinki University Central Hospital, Finland; 4 Department of Public Health, University of Helsinki, Finland; 5 Welfare and Health Policies Division, National Institute for Health and Welfare, Helsinki, Finland; 6 Department of Psychiatry, University of Helsinki, Finland; 7 Institute for Molecular Medicine Finland FIMM, Helsinki, Finland; 8 Department of Social Psychiatry, Tampere School of Public Health, University of Tampere, Finland

## Abstract

**Background:**

Several risk factors for alcohol and other substance use disorders (SUDs) have been identified, but it is not well understood whether their associations with SUD are independent of each other. In particular, it is not well known, whether the associations between behavioral and affective factors and SUDs are independent of other risk factors. The incidence of SUDs peaks by young adulthood making epidemiological studies of SUDs in young adults informative.

**Methods:**

In a comprehensive population-based survey of mental health in Finnish young adults (aged 21-35 years, *n *= 605), structured clinical interview (SCID-I) complemented by medical record data from all lifetime hospital and outpatient treatments were used to diagnose SUDs. We estimated the prevalences of lifetime DSM-IV SUDs, and investigated their associations with correlates from four domains representing: (1) behavioral and affective factors, (2) parental factors, (3) early initiation of substance use, and (4) educational factors. Independence of the association of behavioral and affective factors with SUD was investigated.

**Results:**

Lifetime prevalences of abuse or dependence of any substance, alcohol, and any illicit substance were 14.2%, 13.1%, and 4.4%, respectively. Correlates from all four domains were associated with SUD. The associations between behavioral and affective factors (attention or behavior problems at school, aggression, anxiousness) and SUD were largely independent of other correlates, whereas only daily smoking and low education associated with SUD after adjustment for behavioral and affective factors.

**Conclusion:**

Alcohol use disorders are common in Finnish young adults, whereas other SUDs are less common than in many other developed countries. Our cross-sectional analyses suggested that the association between behavioral and affective factors and SUD was only partly accounted for by other correlates, such as early initiation of substance use and parental alcohol problems. In contrast, associations between many other factors and SUD were non-significant when adjusted for behavioral and affective factors.

## Background

Substance use disorders (SUDs) are among the most common psychiatric disorders and constitute a major public health concern. Recent epidemiological surveys have reported lifetime prevalences of DSM-IV any substance abuse or dependence between 10-20% in the general population [1,2]. Several factors, occurring at the level of individual, interpersonal relations, or society, have been found to increase the risk for SUDs.

A behavioral-temperamental trait often termed disinhibition has been widely recognized as an important risk factor for alcohol and other substance use disorders [3-12]. This trait is characterized by difficulty of inhibiting behavioral impulses, resulting in aggressive or otherwise problematic behavior. Aggression, a key feature in a subtype of conduct disorder and in antisocial personality disorder, is affected by both genetic and environmental factors [13,14]. Childhood aggression predicts substance use problems in adulthood [15], and alcohol abusers often show elevated trait aggressiveness [16].

Besides disinhibitory behavior, also affective traits such as anxiousness may increase the risk for problematic substance use [17]. Mood and anxiety disorders are frequently comorbid with SUDs [18,19], often preceding them, but the processes underlying these associations are not well known [20].

One of the strongest indicators of risk for SUDs is a family history of SUDs. Familial transmission of, and genetic contribution to SUDs are well established [21,22]. Parental SUD also predicts earlier onset of substance dependence in the offspring [23].

The heightened risk related to early onset of substance use is also well established [24]. In addition to being a causal factor, early onset of use may be a marker of pre-existing liability to SUD [25]. Early initiation and heavy smoking have also been found to be risk factors for heavy drinking, and alcohol and other substance use disorders [27,28].

In epidemiological studies, low educational level has consistently been found to associate with SUDs [2,3,11]. Low educational attainment and school problems in adolescence predict substance use and disorders in young adulthood [29]. In addition to own education, parental low education may be related to heavy substance use [30].

Risk factors for SUD are often found to co-occur. For example, parental SUD is associated with behavioral and affective problems in the offspring [31-33], probably accounted for by both genetic and non-genetic familial effects. In addition, both familial alcoholism and disinhibitory traits have been found to predict earlier initiation of use of various substances [23,31,34]. All in all, the the relative importance of different risk factors for SUD and their independence of each other's effects are not well understood.

In the present study, variables representing the four domains of (1) behavioral and affective factors, (2) parental factors, (3) early initiation of substance use, and (4) educational factors were studied as correlates of alcohol and other substance use disorders in young adulthood. As substance use and the incidence of SUDs generally peak around this age [2,35], studying young adults captures most cases within a reasonably short period after disorder onset and minimizes complications arising from the course of the disorder. Using data from a survey representative of the Finnish population, and comprehensive diagnostic assessment, our first aim was to estimate the prevalence of alcohol and other substance use disorders among Finnish young adults. Secondly, we aimed to investigate the relative importance of behavioral and affective factors, parental factors, early initiation of substance use, and educational factors as correlates of SUD, specifically focusing on whether behavioral and affective factors and correlates from other domains associate with SUD independently of each other. Based on previous research, we expected correlates from all the selected domains to individually associate with SUD. Further, we hypothesized that behavioral and affective factors would show strong associations with SUD even when other domains are taken into account, but that associations between many other factors and SUD would be diminished controlling for behavioral and affective factors.

## Methods

### Sample

**Figure 1:**
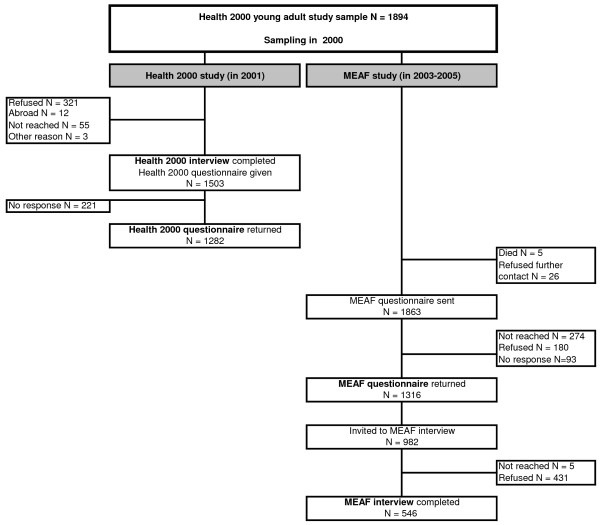
**Sampling and data collection in the Health 2000 and Mental Health in Early Adulthood in Finland (MEAF) studies**.

The data reported here come from a population-based sample of Finnish young adults. The sample was initially assessed in 2001 as part of the nationwide Health 2000 Survey [19,36,37] and re-examined in 2003-2005 to investigate psychiatric disorders among young adults in the Mental Health in Early Adulthood in Finland (MEAF) study [38,39] (Figure 1). MEAF was a two-phase study. In the first phase, a questionnaire was sent to all living members of the original study population who had not refused further contact. In the second phase, persons who were screened positive for mental health or substance use problems, and a random sample of screen-negative persons were invited to a mental health interview.

The MEAF questionnaire included several scales assessing mental health and substance use, to be used as screens for the mental health interview. Two separate screens were used to assess substance use: score of at least three in the CAGE questionnaire [40] for alcohol use, and self-reported use of any illicit drug at least six times. In addition to screen-positive persons, individuals with hospital treatment due to any mental or substance use disorder (ICD Chapter V: Mental and behavioural disorders) during the lifetime according to the Finnish Hospital Discharge Register information were asked to participate in the interview.

Because of the study design, there were non-respondents in two study phases: in the questionnaire containing the screens for the interview, and in the interview. Of the 1863 members of the original study population approached, 1316 (70.6%) returned the questionnaire. Participation in the interview was 55.8% (458/821) for the screen-positive and 54.7% (88/161) for the invited screen-negative persons. Previous analyses indicated that attrition in both study phases was related to age, sex, and education, but not to self-reported mental health disorders or symptoms, including the CAGE scores [38]. Age, sex, and attained education in 2001 were used when calibrating post-stratification weights to correct for non-response.

The study protocol was accepted by the ethics committees of the National Public Health Institute and the Hospital District of Helsinki and Uusimaa. Participants provided written informed consent.

### Alcohol and other substance use disorder diagnoses

The mental health interview was the Research Version of Structured Clinical Interview for DSM-IV-TR [41]. All interviews were conducted by experienced research nurses or psychologists, and were reviewed by the interviewer together with a psychiatrist. For the final diagnostic assessment all case notes from hospital and outpatient treatments were obtained, excluding individuals who had refused any participation in the Health 2000 study. The final best-estimate diagnoses were made by two psychiatrists and two residents in psychiatry. Diagnostic evaluation was based on all available information from the interview and/or case records. All SUDs except for nicotine dependence were assessed.

Diagnostic assessment was completed in 605 individuals (aged 21-35 years), of whom 546 participated in the psychiatric interview and the rest were diagnosed based on case records. The reliability of the diagnoses was tested on 40 cases rated by all four clinicians. For alcohol abuse or dependence, the unweighted kappa values between each pair of raters ranged from 0.94 to 1.00. A detailed description of the methods of MEAF has been provided elsewhere [38]. The present investigation utilized data from both Health 2000 and MEAF studies (Figure 1, Table 1).

**Table 1:** Variables used in logistic regression models, and their origins in different study phases.

Study phase	Variables
Health 2000*	
Questionnaire	Parental alcohol problems
Interview	Attention or behavior problems at school, Parental basic education, Learning difficulties at school
MEAF**	
Questionnaire	Aggression, Anxiousness, Age at initiation of daily smoking, Age at initiation of drinking to intoxication
Interview	SUD diagnoses, Basic education

MEAF, Mental Health in Early Adulthood in Finland

* in 2001

** in 2003-2005

### Behavioral and affective factors

#### Attention or behavior problems at school

A set of questions concerning difficulties during school time, lasting longer than one semester (four to five months), was asked. A positive response to either of the items on attention or behavior problems indicated attention or behavior problems at school.

#### Aggression

A short measure of trait aggressiveness was constructed based on selected items from the Buss-Perry Aggression Questionnaire [42]. Two items from each of the four aggression subscales were translated into Finnish, creating an eight-item scale. A summary scale of the eight items, responded to on a five-point Likert scale, was constructed (theoretical range 8-40, Cronbach's alpha = .82). Aggression scores were further classified as low (<11), moderate (11-17), and high (>17), approximating the observed 25^th ^and 75^th ^percentiles.

#### Anxiousness

Trait anxiousness was measured with a single item, which has been used as a measure of anxiousness in previous studies in Finland [43]. The question asked was "Are you usually tense or distressed?". The five-point scale was: 1 "I have good control over my feelings and do not become tense or distressed easily", 2 "I do not feel tense or distressed", 3 "I become distressed quite easily", 4 "I become anxious, tense or distressed very easily", and 5 "I feel anxious or tense all the time as if I had lost my nerves". A three-class variable was created by classifying anxiousness scores 1 and 2 as low, score 3 as moderate, and scores 4 and 5 as high.

### Parental factors

#### Parental alcohol problems

A series of questions concerning various childhood adversities, experienced before age 16, was asked. Items "Did your father have alcohol problems" and "Did your mother have alcohol problems" were combined so that a positive response to either item was considered as an indicator of parental alcohol problems.

#### Parental basic education

Using the highest secondary educational level of both parents, parental basic education was classified as a binary variable of having at least some high school studies or less than high school.

### Substance use initiation

#### Age at initiation of daily smoking

Lifetime never-smokers formed their own category, while for smokers the age at daily smoking initiation was classified into three classes: 18 years or older, 15-17 years, and younger than 15 years.

#### Age at initiation of drinking to intoxication

The question "At which age were you for the first time so drunk that you felt sick afterwards?" was asked. Three classes were created for the age at initiation of drinking to intoxication: those responding "Never" or at age 18 or older, at age 15-17, and at age younger than 15 years.

### Educational factors

#### Learning difficulties at school

Having had learning difficulties at school was determined as a positive response to any of the four learning related difficulties items (Reading, Writing, Mathematics, Languages) in the set of questions related to school time problems. The variable for learning difficulties at school thus represents learning difficulties in reading, writing, mathematics, or languages (or any combination of these) lasting longer than one semester in elementary school.

#### Basic education

A binary variable for basic education was created coding high school degree and less than high school as separate categories.

### Statistical analysis

The lifetime prevalences of substance-specific abuse and dependence diagnoses and any substance abuse or dependence were estimated separately for men and women. Next, the associations between the selected risk factors and lifetime any substance abuse or dependence were studied, first using t-tests and chi-square tests, and then with a series of logistic regression models. These logistic regression models were designed to provide information on whether behavioral and affective factors and risk factors from other domains associate with SUD independent of each other.

The initial cluster sampling design of the Health 2000 Survey [36] was taken into account in the analyses, and post-stratification weights calibrated by Statistics Finland were used to adjust for non-response. These weights correct the survey distributions to correspond to the population distributions. In addition, the two-phase screening for the MEAF mental health interview was taken into account using expansion weights calculated for the screen-positives (M) by dividing their total by the number interviewed (M1), i.e. M/M1, and for the screen-negatives in the same way, N/N1 [44,45]. These weights were calculated separately for men and women. The final weights used in statistical analyses were obtained by multiplying the expansion weights by the post-stratification weights. The weighting procedure has been described in more detail elsewhere [38]. The statistical analyses were performed using Stata 9 with survey settings [46].

#### Missing data

Data from four distinct sources were utilized in the logistic regression models (Table 1, Figure 1). Of the 546 individuals who participated in the MEAF interview, six were dropped because of missing information in three variables from the MEAF questionnaire (Aggression, Anxiousness, and Age at initiation of drinking to intoxication). In addition, there were five individuals who had responded to seven out of the eight items of the aggression scale in the MEAF questionnaire. For these individuals the mean of the seven existing responses for each individual was substituted for the missing value. Further, in order to use all available information, individuals who had participated in the MEAF interview but had missing data in any of the four variables from the Health 2000 study (Table 1) were also included in the logistic regression analyses by coding missingness as a separate category of these categorical variables [47].

## Results

### Lifetime prevalence of alcohol and other substance use disorders

The lifetime prevalence of any substance abuse or dependence was 14.2% (95% CI: 11.6-17.4%). In general, prevalences were higher in men than in women (for any substance abuse or dependence 20.9% [95% CI: 16.5-26.1%] vs. 7.4% [95% CI: 4.9-10.9%], respectively). Alcohol diagnoses were decidedly most prevalent (13.1%), followed by cannabis (1.7%) and amphetamine (1.5%). The prevalence of opioid dependence was 1.0%, and that of any illicit drug abuse/dependence 4.4% (Table 2). Of the cases with SUD diagnosis, 24% had an abuse or dependence diagnosis in two or more classes of substances. The prevalence of any illicit substance diagnosis without comorbid alcohol diagnosis was 1.1%. In 53% of the cases with SUD the age at onset of abuse/dependence was 18 years or younger.

**Table 2:** Prevalences and 95% confidence intervals (CI) of lifetime substance use disorders among young adults in Finland (n = 605)^a^

	Males		Females		Total	
	
	%	(95% CI)	%	(95% CI)	%	(95% CI)
Any substance* abuse/dependence	20.9	(16.5-26.1)	7.4	(4.9-10.9)	14.2	(11.6-17.4)
Alcohol abuse/dependence	19.8	(15.6-24.8)	6.3	(4.1-9.6)	13.1	(10.5-16.2)
Abuse	11.6	(8.4-15.6)	3.4	(1.8-6.2)	7.6	(5.7-10.0)
Dependence	8.2	(5.7-11.7)	2.9	(1.6-5.3)	5.6	(4.0-7.7)
Cannabis abuse/dependence	2.7	(1.4-5.3)	0.7	(0.2-2.4)	1.7	(1.0-3.1)
Abuse	1.4	(0.6-3.5)	0.6	(0.1-2.3)	1.0	(0.5-2.1)
Dependence	1.3	(0.5-3.4)	0.2	(0.0-1.2)	0.7	(0.3-1.8)
Amphetamine abuse/dependence	1.9	(0.9-4.0)	1.0	(0.3-3.1)	1.5	(0.8-2.7)
Abuse	0.2	(0.0-1.5)	0		0.1	(0.0-0.8)
Dependence	1.7	(0.7-3.8)	1.0	(0.3-3.1)	1.3	(0.7-2.6)
Opioid abuse/dependence	1.3	(0.5-3.4)	0.7	(0.2-2.7)	1.0	(0.4-2.2)
Abuse	0		0		0	
Dependence	1.3	(0.5-3.4)	0.7	(0.2-2.7)	1.0	(0.4-2.2)
Sedative abuse/dependence	1.6	(0.7-3.9)	0		0.8	(0.3-2.0)
Abuse	0.7	(0.2-2.6)	0		0.3	(0.1-1.4)
Dependence	0.9	(0.3-2.9)	0		0.5	(0.2-1.5)
Other substance abuse/dependence	1.6	(0.7-3.6)	0		0.8	(0.4-1.8)
Polysubstance dependence	1.1	(0.4-3.1)	0.9	(0.3-2.8)	1.0	(0.5-2.2)
Drug abuse/dependence	6.8	(4.4-10.2)	2.0	(0.9-4.2)	4.4	(3.1-6.3)

^a ^Calculated using expansion weights

* Excluding tobacco

### Correlates

#### Unadjusted associations

Distributions of age, gender, and correlates from the four domains in people with and without SUD are presented in Table 3. On average, individuals with a SUD diagnosis were older than individuals with no SUD diagnosis [t(538) = -2.9, p < .01], and the male:female ratio was higher in the diagnosis group [χ^2^(1) = 27.9, p < .001]. Individually, all variables from the four domains were significantly associated with SUD (Table 3).

**Table 3:** Differences in covariates and risk factors from four domains between individuals with and without SUD diagnosis (n = 540)

	No SUD diagnosis (n = 464)	SUD diagnosis (n = 76)	t or χ^2 ^(df)	P
**Covariates**				
Age: Mean (SD)	27.9 (3.6)	29.2 (3.7)	-2.88 (538)	<.01
Gender, %				
Female	62.5	30.3		
Male	37.5	69.7	27.85 (1)	<.001
**Behavioral & affective factors**				
Attention or behavior problems at school, %				
No	87.1	43.4		
Yes	7.1	32.9		
Missing	5.8	23.7	80.82 (2)	<.001
Aggression, %				
Low	21.6	13.2		
Moderate	59.7	36.8		
High	18.8	50.0	35.85 (2)	<.001
Anxiousness, %				
Low	77.2	59.2		
Moderate	19.6	26.3		
High	3.2	14.5	21.38 (2)	<.001
**Parental factors**				
Parental alcohol problems, %				
No	66.4	31.6		
Yes	21.8	26.3		
Missing	11.9	42.1	50.56 (2)	<.001
Parental basic education, %				
Some high school	30.4	13.2		
Less than high school	61.4	60.5		
Missing	8.2	26.3	23.92 (2)	<.001
**Age at substance use initiation**				
Smoking, %				
Non-smoker	47.8	13.2		
>17 years	14.7	18.4		
15-17 years	24.8	32.9		
<15 years	12.7	35.5	41.89 (3)	<.001
Drinking to intoxication, %				
>17 years or never	32.5	15.8		
15-17 years	48.5	44.7		
<15 years	19.0	39.5	18.83 (2)	<.001
**Learning & education**				
Learning difficulties at school, %				
No	86.4	56.6		
Yes	7.8	19.7		
Missing	5.8	23.7	42.00 (2)	<.001
Basic education, %				
High school	61.6	23.7		
Less than high school	38.4	76.3	38.23 (1)	<.001

SUD, substance use disorder; SD, standard deviation

Interactions between gender and all correlates were also assessed, and significant interactions between gender and aggression, and gender and parental education (p < .01 in both cases) were observed. All women with SUD scored moderate or high in aggression, whereas one fifth of men with SUD scored low in aggression. The interaction between parental education and gender was due to there being no differences in the distribution of parental education between women with and without SUD, whereas low parental education was more common in men with SUD (χ^2^(2) = 37.6, p < .001).

#### Adjusted associations

Next, a series of logistic regression models was conducted to assess the associations between behavioral and affective factors and SUD adjusting for correlates from other domains. To facilitate interpretation of the models, the unadjusted associations from Table 3 are presented as odds ratios (ORs) in the first column of Table 4. The second column gives the adjusted odds ratios (AORs) for each variable adjusting for the other variables in the same domain, and the third column further adjusts these associations for age and gender.

**Table 4:** Associations (odds ratios) between risk factors from four domains and lifetime any substance abuse/dependence among young adults in Finland (n = 540)^a^

	Univariate		Blocks		Blocks+age & sex		Model I		Model II		Model III		Model IV		Model V	
	OR	(95% CI)	AOR	(95% CI)	AOR	(95% CI)	AOR	(95% CI)	AOR	(95% CI)	AOR	(95% CI)	AOR	(95% CI)	AOR	(95% CI)
**Behavioral & affective factors**																
Attention or behavior problems at school																
No	1		1		1		1		1		1		1		1	
Yes	**11.6**	(5.61-23.97)	**7.0**	(3.27-14.79)	**6.8**	(2.93-15.63)	**6.8**	(2.93-15.63)	**6.0**	(2.53-14.19)	**5.0**	(2.02-12.23)	**4.9**	(1.80-13.48)	**3.4**	(1.13-10.11)
Missing	**11.2**	(5.64-22.15)	**8.3**	(4.21-16.55)	**8.1**	(4.15-15.72)	**8.1**	(4.15-15.72)	2.2	(.20-24.08)	**5.2**	(2.29-11.95)	**6.5**	(3.25-13.11)	1.6	(.24-11.09)
Aggression																
Low	1		1		1		1		1		1		1		1	
Moderate	1.4	(.61-3.34)	1.3	(.56-2.99)	1.6	(.67-3.87)	1.6	(.67-3.87)	1.8	(.74-4.17)	1.4	(.52-3.56)	1.4	(.55-3.37)	1.3	(.51-3.41)
High	**7.6**	(3.59-16.03)	**3.5**	(1.55-7.80)	**4.3**	(1.84-9.90)	**4.3**	(1.84-9.90)	**4.1**	(1.77-9.64)	**3.5**	(1.32-9.44)	**3.0**	(1.21-7.61)	2.7	(.94-7.79)
Anxiousness																
Low	1		1		1		1		1		1		1		1	
Moderate	**2.5**	(1.39-4.54)	1.4	(.69-2.99)	**2.2**	(1.01-4.65)	**2.2**	(1.01-4.65)	2.0	(.92-4.40)	**2.9**	(1.37-6.19)	**2.5**	(1.14-5.40)	**3.0**	(1.33-6.91)
High	**7.7**	(2.87-20.58)	1.8	(.58-5.62)	3.0	(.92-9.98)	3.0	(.92-9.98)	3.2	(.99-10.40)	**3.8**	(1.08-13.65)	3.2	(.86-11.71)	**4.0**	(1.07-15.14)
																
**Parental factors**																
Parental alcohol problems																
No	1		1		1				1						1	
Yes	**2.4**	(1.22-4.76)	**2.2**	(1.09-4.44)	**2.4**	(1.13-5.03)			1.5	(.72-3.30)					1.6	(.73-3.59)
Missing	**6.5**	(3.22-13.05)	**4.3**	(1.75-10.77)	**3.6**	(1.36-9.73)			**3.4**	(1.03-11.58)					2.7	(.82-9.04)
Parental basic education																
Some high school	1		1		1				1						1	
Less than high school	**2.9**	(1.38-6.23)	**2.8**	(1.28-5.93)	**3.0**	(1.35-6.55)			2.3	(.97-5.60)					1.9	(.78-4.76)
Missing	**12.3**	(5.11-29.63)	**5.1**	(1.67-15.77)	**6.8**	(2.06-22.54)			2.8	(.20-39.57)					2.6	(.38-18.43)
																
**Age at substance use initiation**																
Daily smoking																
Non-smoker	1		1		1						1				1	
>17 years	**4.3**	(1.70-11.01)	**4.0**	(1.46-10.89)	**3.7**	(1.36-9.94)					**3.1**	(1.07-9.00)			**3.4**	(1.21-9.51)
15-17 years	**5.0**	(2.10-12.09)	**4.4**	(1.77-10.91)	**4.2**	(1.64-10.82)					**3.4**	(1.29-9.18)			**3.0**	(1.10-8.29)
<15 years	**14.5**	(5.92-35.33)	**8.9**	(3.21-24.90)	**9.9**	(3.37-28.80)					**8.5**	(2.89-25.11)			**7.5**	(2.56-22.19)
Drinking to intoxication																
>17 years or never	1		1		1						1				1	
15-17 years	2.0	(.97-4.05)	1.3	(.61-2.76)	1.4	(.62-3.09)					1.3	(.54-3.12)			1.4	(.58-3.56)
<15 years	**6.7**	(2.94-15.44)	**2.6**	(1.01-6.76)	2.7	(.92-7.66)					2.1	(.76-5.88)			2.2	(.76-6.44)
																
**Learning & education**																
Learning difficulties at school																
No	1		1		1								1		1	
Yes	**3.8**	(1.76-8.03)	**2.9**	(1.33-6.30)	**4.3**	(1.83-9.95)							1.2	(.42-3.21)	1.6	(.52-4.87)
Missing	**8.4**	(4.30-16.35)	**6.5**	(3.34-12.60)	**7.0**	(3.67-13.22)							*		*	
Basic education																
High school	1		1		1								1		1	
Less than high school	**6.4**	(3.64-11.29)	**5.4**	(3.02-9.62)	**4.4**	(2.39-7.99)							**3.1**	(1.60-6.14)	1.8	(.93-3.65)

**Log likelihood of the model**							-332.564		-319.966		-297.473		-320.756		-283.803	
**Likelihood ratio chi2 (df)**^b^									25.196 (4)		70.182 (5)		23.616 (2)		97.522 (11)	
**P value**									<.001		<.001		<.001		<.001	

^a ^Calculated using expansion weights

^b ^Compared to Model I

* Dropped due to collinearity with missingness in Attention or behavior problems at school

In the univariate models the association between each predictor variable and SUD was assessed separately. The column labelled "Blocks" gives results for each variable adjusting for other variables from the same block.

The column labelled "Blocks + age & sex" gives results for each variable adjusting for other variables from the same block plus age and gender In Models I-V the blocks shown were entered in the model, and age and gender were adjusted for. Associations significant at p < .05 or lower are shown in boldface.

OR, odds ratio; AOR, adjusted odds ratio; CI, confidence interval

In *Model I*, behavioral and affective factors and the covariates age and gender were included as predictor variables. When assessed simultaneously, all three variables (attention or behavior problems at school, aggression, and anxiousness) still had significant associations with SUD diagnosis (AORs 2.2-6.8) (Table 4).

*Model I *established the baseline for the effect of behavioral and affective factors, with which the subsequent models could be compared. In *Model II *(Table 4), parental factors were added. The AORs of attention or behavior problems at school and aggression remained significant and the changes in odds ratios were not significant. The effect of high anxiousness almost attained statistical significance (p = .053). Among parental factors only missing information for parental alcohol problems associated with SUD. In *Model III *(Table 4), the effect of early initiation of substance use was assessed. Age at initiation of drinking to intoxication was not associated with risk for SUD, whereas daily smoking was associated with elevated risk. Initiation of daily smoking before age 15 showed a large effect (AOR = 8.5). Behavioral and affective measures remained significant predictors of SUD, but the AOR of attention or behavior problems at school was reduced compared to *Model I *(adjusted Wald test, p = .042). In *Model IV *(Table 4), a similar analysis was conducted with measures of learning and education. Learning difficulties at school showed no risk independent of behavioral and affective factors, but not having a high school degree was associated with SUD (AOR = 3.1). Attention or behavior problems at school, high aggression, and anxiousness still had significant associations with SUD, but the AOR of high aggression was reduced compared to *Model I *(adjusted Wald test, p = .020).

Finally, in *Model V *(Table 4), the correlates from all four domains were assessed simultaneously. Adjusting for all the correlates, the AORs of attention or behavior problems at school and anxiousness remained significant, whereas high aggression failed to reach statistical significance (p = .065). Of the other domains, only age at initiation of daily smoking emerged as a statistically significant correlate. Compared to non-smokers, smokers regardless of the age at initiation were at elevated risk. Having initiated daily smoking before age 15 had a strong association with SUD (AOR = 7.5). We also ran the analyses using the aggression score as a continuous variable, and no significant changes were seen in the results for other variables. The AOR associated with a 1 unit change in aggression in the final model was 1.1 (95%CI: 1.00-1.14, p = .051).

Although the AORs for many variables were nonsignificant in Models II-V, these additional domains of correlates clearly improved the statistical prediction of SUD over behavioral and affective factors only, as is evident from the statistically significantly higher maximum likelihood of these models compared to Model I (Table 4). These comparions take account of the number of additional variables.

## Discussion

### Prevalence of alcohol and other substance use disorders

Using population-based data and comprehensive diagnostic assessment based on structured clinical interview and medical case records, we found that approximately 14% of Finnish young adults had a lifetime SUD, and that an overwhelming majority of the cases were alcohol disorders. In general, the prevalences were higher in men than in women. The estimated lifetime prevalence of any SUD was fairly similar to recent estimates for the US from the National Comorbidity Survey Replication, which reported a lifetime prevalence of 16.7% of any SUD in the age group 18-29 years [2]. On the other hand, The National Epidemiologic Survey on Alcohol and Related Conditions, also from the US, reported substantially higher lifetime prevalences of both alcohol (30.1%) and drug disorders (14.2%) in this age group [3,11] compared to the present results (13.1% and 4.4%, respectively). In Europe, Wittchen *et al*. reported a similar lifetime prevalence of any substance disorder of 17.7% among adolescents and young adults (aged 14-24) [48].

In addition to true differences between populations, discrepancies in prevalence estimates between studies arise due to use of different diagnostic methods. Notably, both structured clinical interview (SCID-I) and medical record data over the participants' lifetime were used in the diagnostic assessment in the present study. This method was chosen to improve the assessment of clinical significance of the symptoms of mental disorders, which has been deemed a potential problem in psychiatric epidemiological studies [49]. Similar diagnostic assessment methodology was used previously in an epidemiologic study of psychotic disorders in Finland [50].

Information on the lifetime prevalence of alcohol and other substance use disorders among young adults in Finland has not been previously available. Pirkola *et al*. reported the lifetime prevalence of alcohol dependence of 7.9% in the Health 2000 adult sample (aged 30 years and over) [51], whereas in the present sample of young adults the lifetime prevalence of alcohol dependence was 5.6%. In an urban sample of 20-24-year-old Finns, Aalto-Setälä *et al*. estimated one-month prevalence of any SUD to be 6.2%, but the sample only contained cases of alcohol and cannabis disorders [52]. The estimated prevalences of alcohol and other substance use disorders in young adults in the present study fit well with the known profile of substance use in the Nordic countries, characterized by a high level of drinking to intoxication and a fairly low level of use of substances other than alcohol [53-56]. For example, in our study, 75% of young adults reported having been drunk within 12 months, while only 8% reported lifetime use of cannabis for more than five times.

### Correlates of SUDs

#### Unadjusted associations

Our findings replicated previous results of disinhibitory and affective traits as correlates of SUD [7,8,20]. The association between parental alcohol problems and SUD in the offspring was also expected due to the strong familial pattern of substance use problems [21,57]. The effect of parental education is less well studied, but our results point to the possibility of elevated risk for SUD related to low parental education. The finding that early initiation of drinking to intoxication was strongly associated with SUD was anticipated [24], but somewhat surprising was the even stronger association between early onset of smoking and SUD, albeit evidence for the effect of early onset smoking on alcohol and drug disorders has been reported previously [26,28,58]. The observed association between own low education and SUD was not surprising on the grounds of previous studies [2,3,11], but the predictive value of learning difficulties has not been widely studied.

Majority of the studies looking into risk factors for SUDs have been conducted in Anglo-Saxon societies (e.g. refs. [7,8,29,32,34,58,59]). However, the availability of substances and the prevailing general culture of substance use potentially influence the associations between risk factors and SUDs. Thus, it is of importance that the correlates studied here, selected on the basis of previous research, were associated with SUD also in the present sample of young adults from Finland. This finding suggests that the importance of cultural factors notwithstanding, these factors are related to SUDs despite varying cultures of alcohol and other substance use. Further, in an earlier study on this sample, we found several of the correlates reported here to be related to poorer cognitive functioning observed in young adults with SUDs [39].

#### Adjusted associations

Although the measures of attention or behavior problems at school, trait aggression and anxiousness were conceptualized as belonging to the same class of phenomena, our results suggest that they associate with SUD partly independently of each other. This result indicates that in addition to disinhibitory traits, also trait anxiety is strongly associated with elevated risk for SUD. Whether the relationship between anxiety and SUD is causal or reflects shared underlying vulnerability, is currently unknown [20]. It should also be noted that our measure of trait aggression did not assess actual aggressive behavior, but should be regarded as an index of a disposition to experience aggressive feelings.

In their longitudinal study from New Zealand, Fergusson *et al*. assessed the effect of childhood and adolescence conduct and attentional problems on later substance use, abuse and dependence, controlling for various social, family and individual covariates [8]. They found that conduct problems in adolescence were significantly related to use, abuse and dependence of various substances even when the wide array of covariates was included in the model. Another recent longitudinal study also found various self-reported problem behaviors in adolescence to predict SUDs in early adulthood, controlling for maternal education and alcohol use among other factors [60]. Our cross-sectional results, suggesting that the association between behavioral and affective factors and lifetime SUD diagnosis is largely independent of factors related to parents, early initiation of tobacco and alcohol use, and education and learning, are well in line with the findings of these studies. Taken together, these results emphasize the importance of disinhibitory and affective factors associated with SUDs.

Our results suggest that the associations of parental alcohol problems and low basic education with SUD in the offspring are at least partly mediated by the offspring's attention or behavior problems at school, aggression and anxiousness. This interpretation is based on results from Model II, where the previously significant associations between parental factors and SUD were rendered nonsignificant by behavioral and affective factors in the model. This finding is compatible with previous studies reporting that the effect of parental substance use problems on SUDs in the offspring is partly explained by the offspring's disinhibitory traits [59]. Interestingly, a twin study utilizing a children-of-twins design found evidence for the hypothesis that paternal SUD and disinhibitory traits in the offspring have a partly shared genetic background [61].

Smoking increased the odds for SUD irrespective of the age at initiation, but the risk related to daily smoking initiation before age 15 was remarkably elevated. It is well known that cigarette smoking often predates alcohol and drug use, but the meaning of this observation is debated. Our results extend previous findings that age at smoking initiation is a risk factor for SUD independent of family history of alcoholism [26,58] by providing evidence that the association between early smoking initiation and SUD is not accounted for by behavioral and affective factors, parental factors, age at initiation of alcohol use, learning difficulties or lower education. In contrast, the association between early initiation of drinking to intoxication and SUD may be accounted for by smoking and gender, as this association was non-significant when adjusted for these factors.

The relationship between behavioral and affective factors and educational factors was of special interest in the present study. As attention or behavior problems at school are bound to be associated with the level of achieved education, it is noteworthy that our results suggested that the association between low educational level and SUD is only partly accounted for by behavioral and affective factors. Epidemiological surveys have frequently identified low education as a risk factor for SUD [2,3,11], but these studies rarely include other than sociodemographic factors. Our results suggest a relationship between lower education and SUD even when behavioral and affective factors and learning difficulties are taken into account. The temporal nature of this relationship between SUD and education cannot be determined in the present study, but in more than half of the cases the age at first abuse or dependence diagnosis was younger than 19, suggesting that in these cases problematic substance use predated or took place simultaneously with the processes leading to lower educational level. In contrast, learning difficulties did not have an association with SUD independently of behavioral and affective factors. We thus failed to replicate a previous finding that learning difficulties would increase the risk for SUD independently of behavior problems [62]. However, in the Beitchman *et al*. study learning difficulties were objectively tested for, whereas in the present study they were retrospectively self-reported.

### Limitations

The present findings should be considered in conjunction with several limitations. First, as the data are cross-sectional, no conclusions about causality between the measures can be made. Secondly, the variables used in this study were self-reported, and the possibility that those reporting more problematic substance use would be prone to report higher (or lower) levels of other negative factors cannot be excluded. However, it should be noted that several measures of the present study come from a general health survey, conducted at least two years before the psychiatric assessment and not profiled as focusing specifically on SUDs, which should reduce bias in reporting. Third, the assessment of the four domains of correlates of SUD can in no case be considered comprehensive. For example, the single item used to assess anxiousness arguably provides a very limited assessment of affective factors. A fourth limitation has to do with missing data in the Health 2000 variables. In some logistic regression models missingness indicated elevated risk for SUD, reflecting the difficulty of studying individuals with serious substance use problems. However, when all the correlates were assessed, missingness ceased to have a significant effect. In any case, the effect of missing data should be taken into account when assessing the results related to these variables. A further limitation is that it was not feasible to study men and women separately. Significant interactions between gender and two risk factors were observed, but because of the small number of women with SUD in the sample, these interaction terms were not included in the multiple logistic regression models. In addition, due to the small number of other than alcohol disorders, substance specific correlates could not be assessed. A final limitation concerns attrition. As explained in the Methods, due to the two-phase study design, there were non-respondents in both of the study phases. However, non-response was not related to self-reported mental health or alcohol use problems [38]. To statistically correct for non-response, post-stratification and expansion weights were used both in estimating the prevalences and in the logistic regression models.

## Conclusion

Prevalence of lifetime any substance use disorder is approximately 14%, and alcohol disorder approximately 13% among Finnish young adult population, illicit substance use disorder without an alcohol disorder thus being very rare. Behavioral and affective factors, parental factors, early initiation of substance use, and learning difficulties and lower education were all found to be associated with alcohol and other substance use disorders. The association with behavioral and affective factors was only partly accounted for by other correlates. In contrast, only daily smoking and lower education associated with SUD when behavioral and affective factors were taken into account. Associations between many risk factors and SUD may be reflections of behavioral and afffective factors.

## Competing interests

The authors declare that they have no competing interests.

## Authors' contributions

JS, AT-H, JP, SIS, TA-S, HA, SK and JL contributed to the conception and design of the MEAF study. SK and JS also contributed to the conception and design of the baseline survey (Health 2000). JS, AT-H, JP, SIS and TA-S contributed to acquisition of data. AL planned and undertook the statistical analyses, and wrote the first draft of the manuscript. JS, AT-H, SK, TK and JK contributed to the design and discussion of the analyses. All authors participated in revising the manuscript for intellectual content by providing critical comments and have approved the final manuscript.

## Pre-publication history

The pre-publication history for this paper can be accessed here:

http://www.biomedcentral.com/1471-244X/9/73/prepub
